# Tuning the Synthesis of Manganese Oxides Nanoparticles for Efficient Oxidation of Benzyl Alcohol

**DOI:** 10.1186/s11671-016-1777-y

**Published:** 2017-01-06

**Authors:** Jingyuan Fei, Lixian Sun, Cuifeng Zhou, Huajuan Ling, Feng Yan, Xia Zhong, Yuxiang Lu, Jeffrey Shi, Jun Huang, Zongwen Liu

**Affiliations:** 1 School of Chemical and Bimolecular Engineering, The University of Sydney, Sydney, NSW 2006 Australia; 2 School of Materials Science and Engineering, Guilin University of Electronic Technology, Guilin, 541004 China; 3 School of Environment, Tsinghua University, Beijing, China

**Keywords:** Manganese oxides, Synthesis–structure–catalysis interaction, Aerobic oxidation, Benzyl alcohol

## Abstract

The liquid phase oxidation of benzyl alcohol is an important reaction for generating benzaldehyde and benzoic acid that are largely required in the perfumery and pharmaceutical industries. The current production systems suffer from either low conversion or over oxidation. From the viewpoint of economy efficiency and environmental demand, we are aiming to develop new high-performance and cost-effective catalysts based on manganese oxides that can allow the green aerobic oxidation of benzyl alcohol under mild conditions. It was found that the composition of the precursors has significant influence on the structure formation and surface property of the manganese oxide nanoparticles. In addition, the crystallinity of the resulting manganese nanoparticles was gradually improved upon increasing the calcination temperature; however, the specific surface area decreased obviously due to pore structure damage at higher calcination temperature. The sample calcined at the optimal temperature of 600 °C from the precursors without porogen was a Mn_3_O_4_-rich material with a small amount of Mn_2_O_3_, which could generate a significant amount of ${\mathrm{O}}_2^{-}$ species on the surface that contributed to the high catalytic activity in the oxidation. Adding porogen with precursors during the synthesis, the obtained catalysts were mainly Mn_2_O_3_ crystalline, which showed relatively low activity in the oxidation. All prepared samples showed high selectivity for benzaldehyde and benzoic acid. The obtained catalysts are comparable to the commercial OMS-2 catalyst. The synthesis–structure–catalysis interaction has been addressed, which will help for the design of new high-performance selective oxidation catalysts.

**Electronic supplementary material:**

The online version of this article (doi:10.1186/s11671-016-1777-y) contains supplementary material, which is available to authorized users.

## Background

The liquid phase oxidation of benzyl alcohol is a widely investigated reaction by both academics and engineers as it provides benzaldehyde and benzoic acid [1]. Both products can act as versatile intermediates for the synthesis of fine chemicals or perfume [2–7]. In the past few years, numerous catalytic systems have been developed to perform this chemo-selective oxidation of alcohols [6]. Both homogeneous and heterogeneous catalysts can perform this benzyl alcohol oxidation. Traditional homogeneous catalytic systems for the oxidation of benzyl alcohols use stoichiometric chemicals such as chromium (VI) reagents, dimethyl sulfoxide, hydrogen peroxide, tert-butyl hydroperoxide (TBHP), nitric acid, and permanganates or other inorganic oxidants [6, 8]. Homogeneous catalysis normally has excellent reactivity and selectivity in a small scale reaction since in homogeneous catalysts every single catalytic entity can act as a single active site [9–12]. But on an industrial scale and considering environmental and ecological issues, the drawback of homogeneous catalysis is evident, such as catalyst leaking, poisoning, toxic metal waste, difficulty in separation, and recovery of the catalyst from the reaction mixture as well as limited recyclability [12–16].

Hence, heterogeneous catalysts are considered to be a promising alternative for the oxidation of benzyl alcohol, and in fact, heterogeneous catalysis participates in almost 90% of industrial practices currently [15]. Recently, a large number of studies on the oxidation of benzyl alcohol to benzaldehyde by molecular oxygen with a solvent, using various transition metal oxide-based and noble metal-based catalysts, have been reported [14, 17]. The noble metal-based catalysts, though highly active for the oxidation of benzyl alcohol, generally suffer from the sintering of surface noble metal particles and high price as well as a low reserve on earth [18]. Although some advancement has been made in the catalytic oxidation of benzyl alcohols over noble metal catalyst, there are only limited literature reports on non-noble metal catalysts, like transition metals or transition metal oxides especially manganese oxides [19]. From the viewpoint of green economy and environmental demand, it is highly attractive to develop new and cost-effective catalysts using non-precious metals or metal oxides such as nickel [20], vanadium [21], copper [3], and manganese [19] that can allow more efficient aerobic oxidation of benzyl alcohol under mild conditions [14].

Among most of transition metal oxides, manganese oxides attract a broad interest, as they can exist in more than five easily exchangeable transition states with various structural forms over a wide range of temperatures (up 1200 °C) [22–24]. Manganese oxides have various catalytic applications due to highly efficient redox properties, and the mixed valence has been confirmed to be important in redox catalysis as well as in energy and electron transfer [23, 25, 26]. Tu and co-workers demonstrated that manganese oxides (II, III, IV, and VII) have significant advantages over other oxidants in removing organic pollutants [25]. Furthermore, it has been confirmed that reduced manganese oxide can readily be reoxidized by dioxygen, meaning that manganese oxide can play as an electron-transfer mediator to generate a rapid electron-transfer path during oxidation reactions [27]. Many factors can affect the performance of manganese oxide catalysts during the oxidation of benzyl alcohols, but there were only a few studies exploring the influence of calcination temperatures of precursors on the physiochemical property and catalytic activity of the final products [28]. Furthermore, plenty of materials with similar gross structure features might have various properties due to different particle sizes and the amount and type of defects formed during different synthesis procedures. So even slight changes of synthetic parameters can result in distinct properties in catalytic, electrochemical, or ion-exchange activity [22]. In this report, transition metal-manganese oxide nanoparticles have been prepared by the variation of precursors and thermally controlled calcination. Their catalytic performance has been addressed by the oxidation of benzyl alcohol under the liquid phase with oxygen. The correlation among synthesis conditions, structure, and catalysis has been formulated based on the characterization and experimental results.

## Methods

### Catalyst Synthesis

Absolute ethanol, ammonia solution, analytical-grade manganese (II) chloride tetrahydrate, dopamine hydrochloride, and Pluronic® F127 were all purchased from Sigma-Aldrich and used without further purification.

The volumes of 100 mg F127 (7.94 × 10^−7^ mol), 100 mg dopamine hydrochloride (5.27 × 10^−4^ mol), and 60 mg manganese (II) chloride tetrahydrate (3.03 × 10^−4^ mol) were dissolved in a mixture of 10 ml deionized water and 20 ml absolute ethanol. The final mixture was stirred for 2 h (500 rpm) to allow complete dissolution. Then, 0.4 ml ammonia solution was dropwise added into the mixed solution. After stirring for 24 h, the obtained precipitate was sequentially separated from the solution via centrifugation and washed with deionized water and ethanol, and then the black powder was dried at 80 °C overnight in the oven. This was the sample preparation procedure for precursor S1. Totally, three series of samples were made using the control variable method (Table 1).

**Table 1:** Sample compositions

Sample series	Polymer	Porogen	Mn source
S1	PEO_106_–PPO_70_–PEO_106_ (F127)	C_6_H_3_(OH)_2_–CH_2_–CH_2_–NH_2_ (DA)	MnCl_2_·4H_2_O
S2		C_6_H_3_(OH)_2_–CH_2_–CH_2_–NH_2_ (DA)	MnCl_2_·4H_2_O
S3	PEO_106_–PPO_70_–PEO_106_ (F127)		MnCl_2_·4H_2_O

**Fig. 1:**
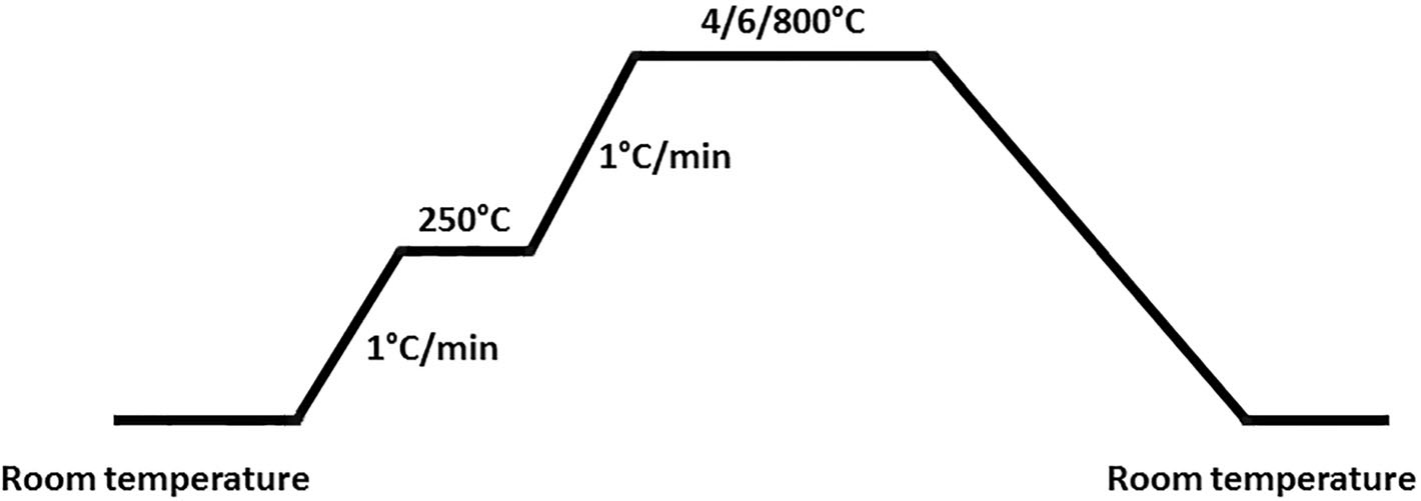
Heating procedure of catalyst precursor

The obtained precursors (S1–S3) were thermally treated at temperatures of 400, 600, and 800 °C in air according to the heating procedure as shown in Fig. 1.

### Catalyst Characterization

The crystal structures of the prepared samples were characterized by X-ray diffraction (XRD) on a XRD Siemens D5000 using Cu Kα irradiation with a maximum power of 2200 W. The wide-angle XRD patterns were collected over a 2*θ* range of 10–80° with a scan step rate of 2°/min and 0.02°/step. The surface area, pore volume, and pore size distribution were characterized by liquid nitrogen adsorption/desorption experiments, performed with a Quantachrome Autosorb-iQ automated adsorption system. The samples were degassed at 150 °C for 12 h under helium prior to each measurement. The surface areas were calculated using the Brunauer–Emmett–Teller (BET) method, and the pore sizes and pore volumes were calculated from the desorption branch of the isotherm using the Barrett–Joyner–Halenda (BJH) method. The manganese oxides was observed by a Zeiss Ultra Plus scanning electron microscope using SEM. High-resolution transmission electron microscopy (HR-TEM) images were recorded on a JEOL 2200FS transmission electron microscope at 200 kV. Each sample was suspended in ethanol, and then a drop or two of the suspension was loaded onto a carbon-coated copper grid and allowed to fully dry. Temperature-programmed reduction (TPR) experiments were conducted to get the sample oxidation reduction profiles. The samples were treated with N_2_ for 2 h at 150 °C before each experiment. In the experiments, 5% H_2_ in helium was used at a flow rate of 40 ml/min in the temperature range of 150–900 °C at a ramp of 10 °C/min. Temperature-programmed desorption (TPD) experiments were also done to investigate the surface oxygen activity of the materials in the oxidation reaction using N_2_ as carrier gas.

### Catalyst Activity Measurements

In this benzyl alcohol oxidation procedure, 5 ml benzyl alcohol (0.1 M in deionized water) and the catalyst (50 mg) were put in an autoclave reactor under vigorous stirring (around 700 rpm). The sealed reactor was purged by 3 bar molecular oxygen before heating to the reaction temperature. Sampling was taken at every 2 h, and the product mixture was separated from nanocatalysts by centrifugation. Shimazu Ultra 2010 GC-MS and Plus 2010 GC have been used to analyze the products with octane as an internal standard. The conversion of benzyl alcohol (BA) was calculated by the mole ratio of the disappeared BA during the reaction to the total amount of BA before the reaction. The selectivity of the product was obtained by the mole ratio of the produced chemical to the disappeared BA.

## Results and Discussion

### Structural Characterization of the Manganese Oxides

**Fig. 2:**
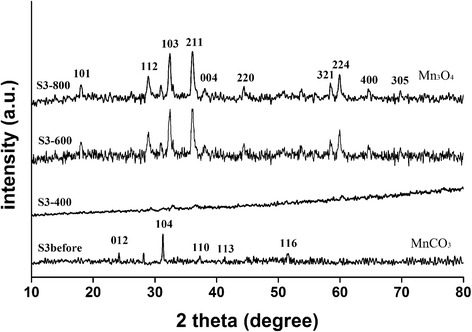
XRD pattern of manganese precursor before and after calcination

The surface areas of the manganese oxides prepared with various precursors and calcined at 400, 600, and 800 °C have been listed in Table 2. Along with the increasing of calcination temperature, the surface area was tapering down. Especially for samples S1 and S2, the surface areas dropped down distinctly, while for S3, the increase of the calcination temperature seemed to not have much influence on the surface area. When looking at the compositions of precursors S1 and S2, although the addition of porogen generated larger surface pores (Fig. 5), it also made the precursors unstable and easier to collapse during calcination and then the surface area decreased significantly. The addition of porogen had remarkable effect on the crystallization for precursors S1 and S2 at the calcination temperature 800 °C, which transformed to bixbyite Mn_2_O_3_ (Additional file 1: Figure S1 and Additional file 2: Figure S2) phase, while S3 turned into hausmannite (Mn_3_O_4_, Fig. 2) phase.

**Table 2:** Catalytic performance of aerobic oxidation of benzyl alcohol over various samples

Entry	Catalyst	BET surface area (m^2^ g^−1^)	Conversion (%)	Selectivity (%)	
				Benzaldehyde	Benzoic acid
1	S1-400	25.7	6.8	100	0
2	S1-600	16.3	49.8	100	0
3	S1-800	13.5	34.7	100	0
4	S2-400	28.3	7.8	100	0
5	S2-600	22.1	62.0	100	0
6	S2-800	11.7	34.2	100	0
7	S3-400	38.6	32.1	100	0
8	S3-600	35.6	64.5	91.8	8.2
9	S3-800	32.2	40.9	100	0

Reaction conditions: 5 ml benzyl alcohol (0.1 M in deionized water), 50 mg catalyst, *T* = 140 °C, 3 bar molecular oxygen, reaction time 5 h

**Fig. 3:**
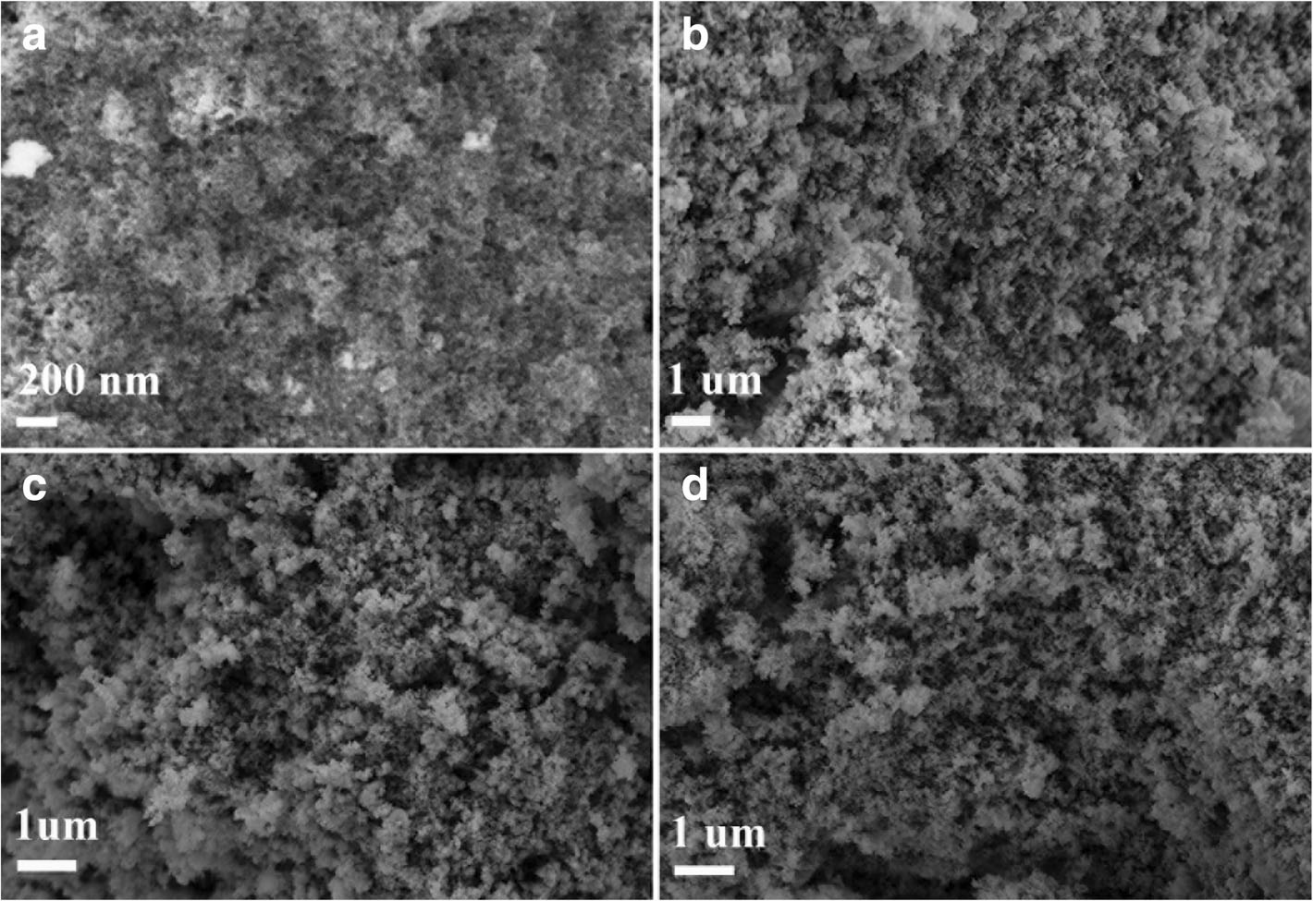
**a**–**d** SEM images of manganese precursor S3 calcined at 400, 600, and 800 °C

The peaks of S3 before calcination (Fig. 2) were similar to the patterns of samples S1 and S2 before calcination (Additional file 1: Figure S1 and Additional file 2: Figure S2). This demonstrates that the prepared manganese precursor was crystalline MnCO_3_. The patterns of S3-600 and S3-800 in Fig. 2 have three main characteristic peaks, indicating the tetragonal crystalline structure of hausmannite Mn_3_O_4_, i.e., (103), (211), and (224), can be observed in both of the XRD patterns (PDF#75-1560). However, the S3 transformed into an amorphous phase after calcination at 400 °C and the other two precursors, S1 and S2, transformed to the body-centered cubic bixbyite Mn_2_O_3_ (PDF#76-0150) (Additional file 1: Figure S1 and Additional file 2: Figure S2) after calcination. Based on the Debye–Scherrer equation analysis of the peak width at half height of sample S3-600 and S3-800 in Fig. 2, the size of the obtained particles was estimated to be around 20 nm, which was confirmed by observing the particle size in the SEM image (Fig. 3).

The SEM images of S3 indicated that there was no obvious morphology change before and after calcination of the precursor except there were more pores formed after calcination. Besides, compared to the SEM images of S1 and S2 (Additional file 3: Figure S3 and Additional file 4: Figure S4), no cubes can be observed in Fig. 3.

**Fig. 4:**
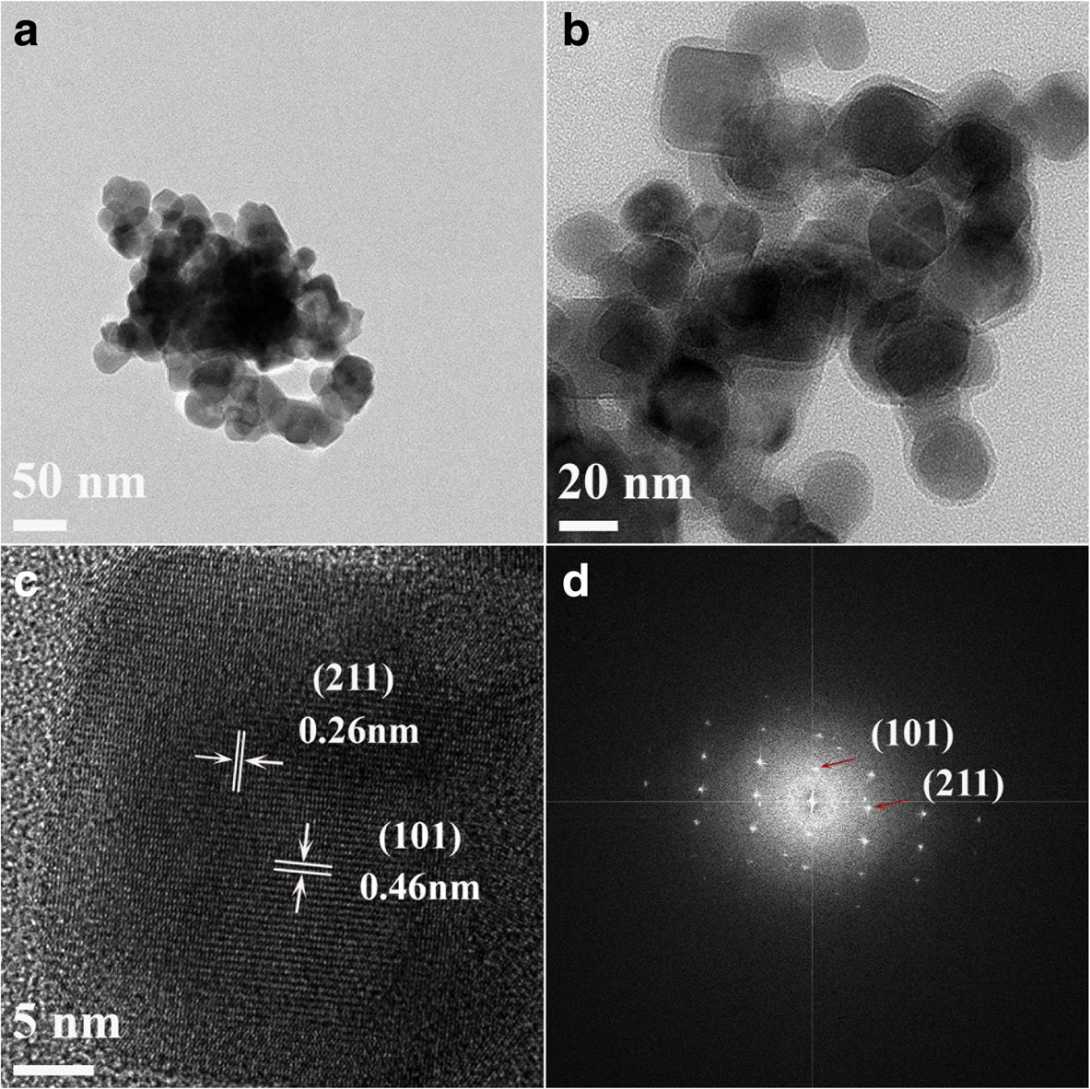
**a**–**d** HR-TEM images of manganese precursor S3 calcined at 600 °C

High-resolution transmission electron microscope (TEM) images of the S3 calcined at 600 °C in air are displayed in Fig. 4. Obviously, the manganese oxide nanoparticles (Fig. 4a, b) had an average size between 20 and 30 nm, and the shape of the particle was more like a regular sphere compared to the oval-shaped particles of S1 and S2 after calcination (Additional file 5: Figure S5 and Additional file 6: Figure S6). The two interplanar spacings of 0.46 and 0.26 nm (Fig. 4c) correspond to the (101) and (211) lattice planes of the tetragonal phase Mn_3_O_4_, respectively. The highly clear crystal lattice as well as the corresponding well-ordered dot pattern of the fast Fourier transform (FFT) image demonstrates the high-quality single-crystalline nature of Mn_3_O_4_.

**Fig. 5:**
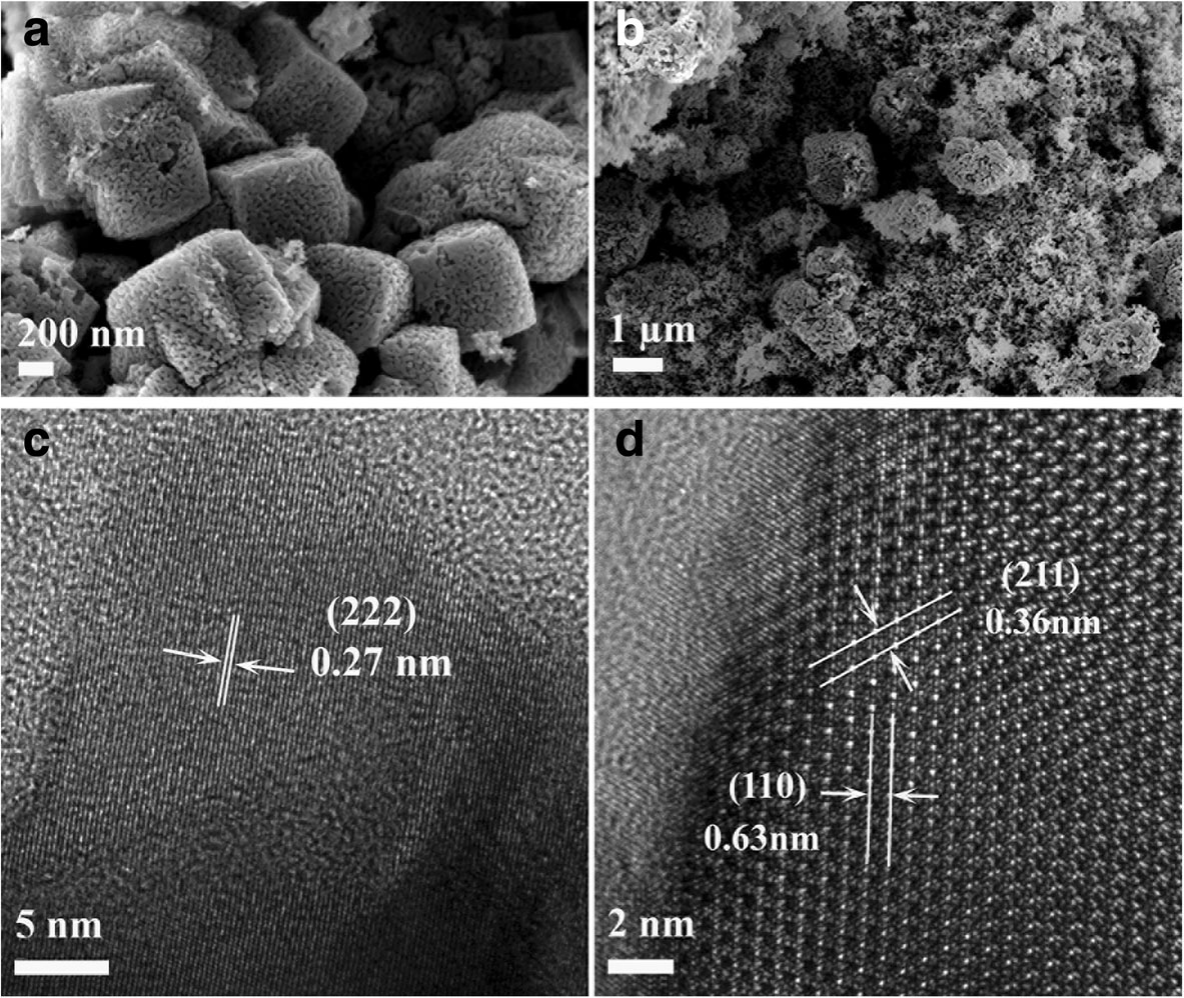
SEM images of precursor S1 (**a**) and precursor S2 (**b**) calcined at 600 °C in air and their corresponding HR-TEM images (**c**, **d**)

After calcination at 600 °C in air, the morphologies of precursor S1 and precursor S2 (Fig. 5) were apparently different from that of precursor S3. Mesopores have been observed in the cubic particles, and all the lattice planes in Fig. 5c, d can be indexed to bixbyite Mn_2_O_3_. Therefore, crystalline Mn_3_O_4_ was formed for S3 and crystalline Mn_2_O_3_ was formed for S1 and S2 after the 600 °C calcination. The compositions for the preparation of precursors had significant influence on the structure formation of manganese oxide nanoparticles.

**Fig. 6:**
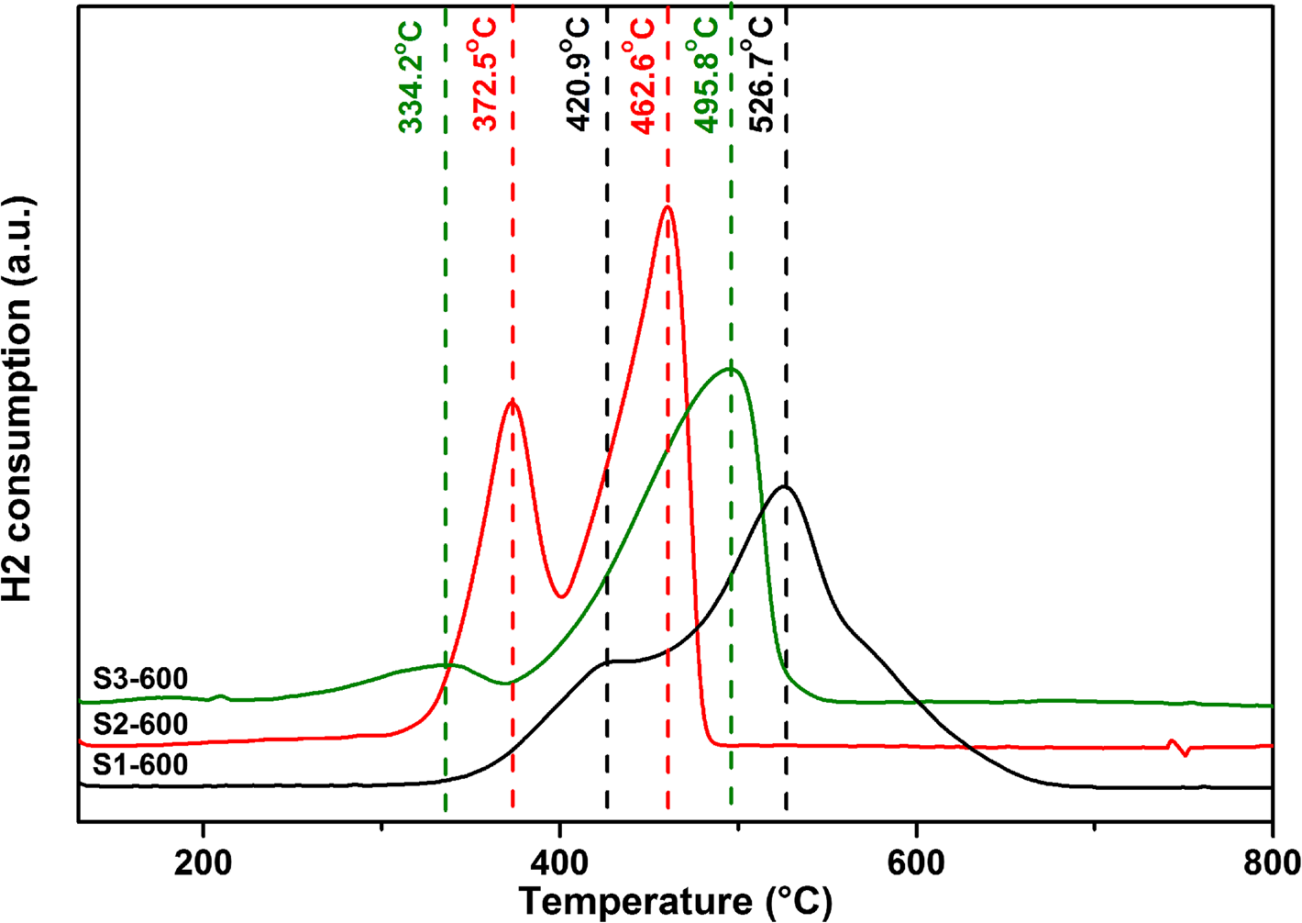
H_2_-TPR profiles of (*black line*) S1-600, (*red line*) S2-600, and (*green line*) S3-600 catalysts heated in a stream of H_2_

The reduction profiles of the materials calcined at 600 °C were tested by the temperature-programmed reduction (H_2_-TPR) studies. From the H_2_-TPR profile of S2-600 as shown in Fig. 6, two reduction peaks can be observed at 372 and 462 °C, indicating a two-step reduction (Mn_2_O_3_ to Mn_3_O_4_ and Mn_3_O_4_ to MnO). The high-temperature reduction peak appeared at 495 °C for S3-600 and 526 °C for S1-600, indicating the difficulty of reducing S3-600 and S1-600 (Mn_3_O_4_ to MnO) comparing to S2-600 (462 °C). However, the low-temperature reduction peak of S3-600 (Mn_2_O_3_ to Mn_3_O_4_) occurred at 334 °C, which was much lower than those of S1-600 and S2-600 (420 and 372 °C, respectively), suggesting the easily reducible nature of S3-600 (from Mn_2_O_3_ to Mn_3_O_4_). According to the XRD and HR-TEM results, S3-600 was a Mn_3_O_4_-rich material, which only contained a trace amount of Mn_2_O_3_ as indicated by a very weak reduction peak at low temperature, while S1-600 still contained a considerable amount of Mn_2_O_3_.

**Fig. 7:**
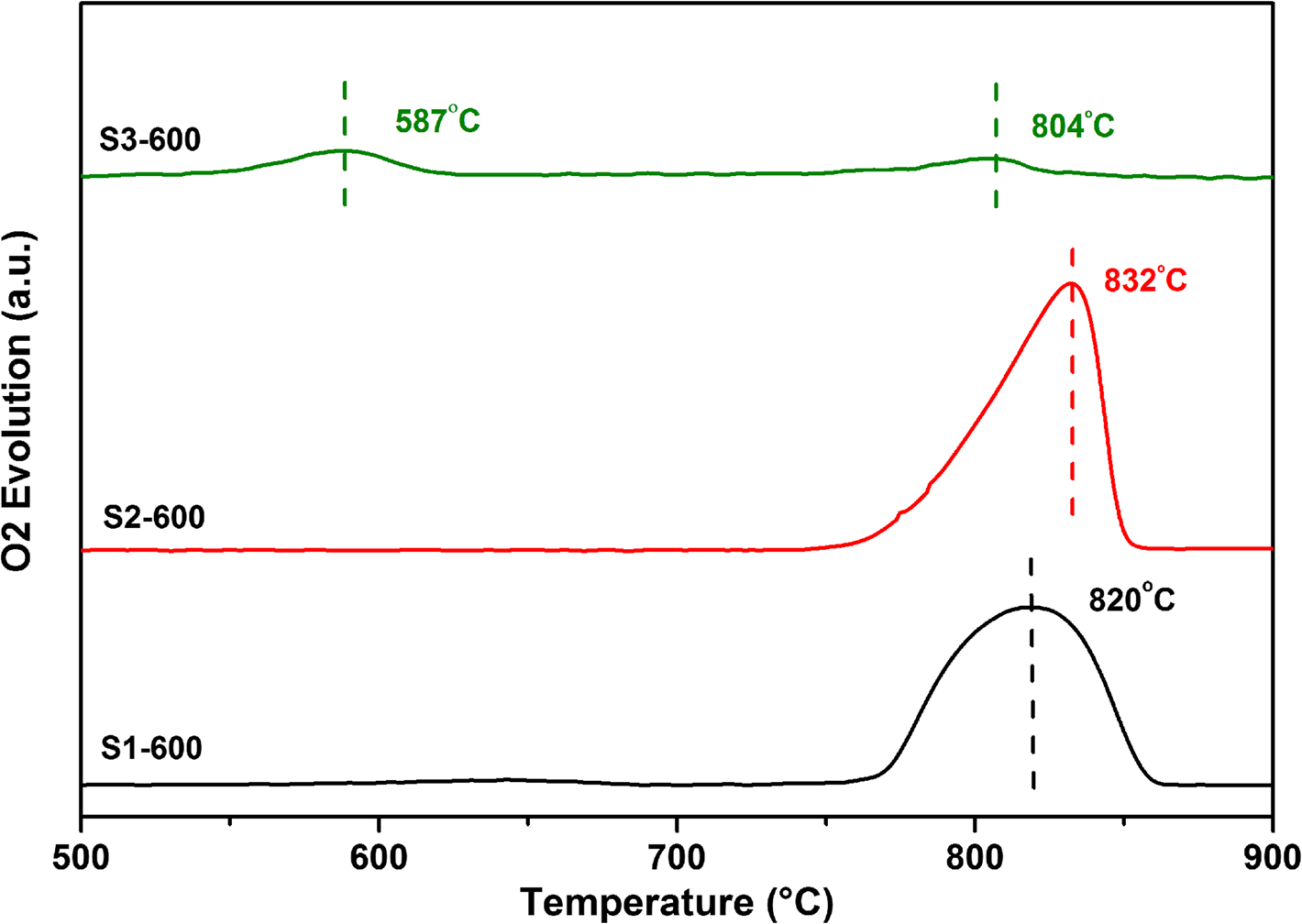
O_2_-TPD profiles of (*black line*) S1-600, (*red line*) S2-600, and (*green line*) S3-600 catalysts heated in a stream of N_2_

The temperature-programmed desorption of O_2_ (O_2_-TPD) was performed to probe the surface active sites of the catalysts for oxygen adsorption/desorption. A major O_2_ loss from both S1-600 and S2-600 was observed at temperatures higher than 800 °C (820 °C for S1-600 and 832 °C for S2-600) in the O_2_-TPD (Fig. 7), which can be ascribed to the loss of lattice oxygen O^2−^, very strong chemisorbed O^−^, or reticular oxygen (known as β-oxygen) corresponding to different Mn–O bond strengths [8, 29–32]. For S3-600, a new and relatively strong desorption peak was observed at 590 °C due to the desorption of active chemisorbed oxygen (species ${\mathrm{O}}_2^{-}$) on the surface with the remarkably enhanced oxygen mobility [7, 33]. Only a slightly small peak at 800 °C was observed for S3-600. Lattice oxygen O^2−^, surface O^−^, or β-oxygen were mainly related to partial oxidation, while the active chemisorbed oxygen ${\mathrm{O}}_2^{-}$ was very active in the deep oxidation [31]. S3-600, a Mn_3_O_4_-rich material with a small amount of Mn_2_O_3_, could generate significant amount of ${\mathrm{O}}_2^{-}$ species on the surface during oxidation that would be responsible for the deep oxidation of alcohols. S1 and S2-600 that were mainly crystalline Mn_2_O_3_ could contain the dominant lattice oxygen O^2−^, surface O^−^, or β-oxygen species on the surface for the partial oxidation.

### Catalytic Reactions

The benzyl alcohol oxidation activities of all samples obtained at different calcination temperatures were studied under identical conditions [14]. Their catalytic performances are listed in Table 2 with their surface areas.

All prepared samples exhibited target selectivity for benzyl alcohol oxidation, with only benzaldehyde and benzoic acid produced. No over oxidation occurred and no CO or CO_2_ was observed by GC/TCD. As shown in Table 2, it is clearly illustrated that samples calcined at 400 °C did not have much oxidation activity as the conversion was quite low. Although samples calcined at 800 °C performed better than those calcined at 400 °C, their conversions were still lower than samples calcined at 600 °C. So in general, the relationship between oxidation activity and calcination temperature followed the order of 400 °C < 800 °C < 600 °C. The oxidation activities of the samples were not increased proportionally with the surface area. For samples calcinated at 400 °C with the highest surface areas, no crystalline Mn oxides were generated resulting in the lowest catalytic activity. Therefore, the oxidation activity should be based on both crystal structure and surface sites.

**Fig. 8:**
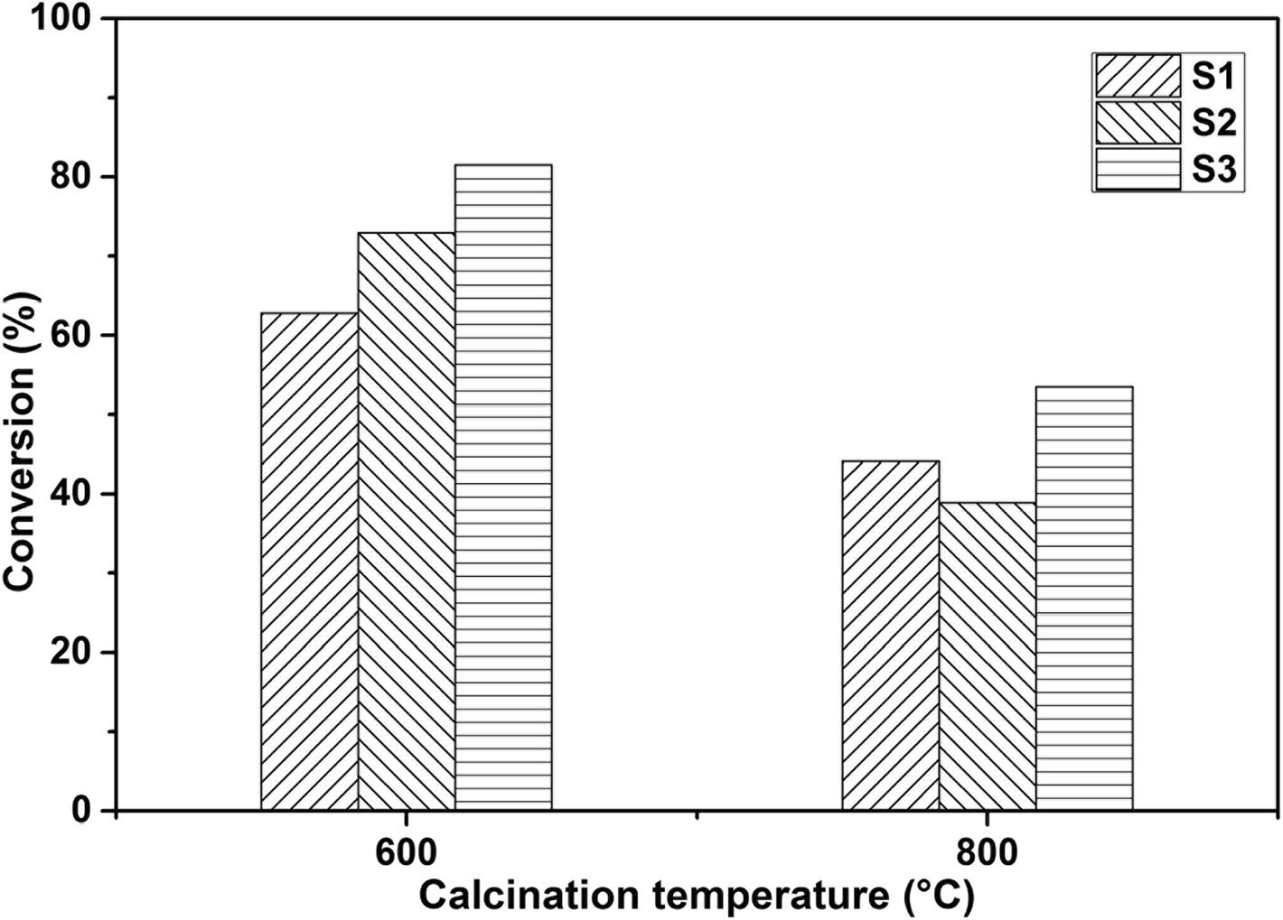
Influence of sample calcination temperatures on benzyl alcohol conversion to benzaldehyde and benzoic acid over catalysts S1, S2, and S3 calcined at 600 and 800 °C, respectively; reaction time 10 h

Among samples calcined at 600 °C, S3-600 performed the best compared to S1-600 and S2-600 in 5 h under the same conditions as shown in Table 2. Figure 8 illustrates benzyl alcohol conversion over precursors S1–S3 calcined at 600 and 800 °C after 10-h reaction as the reaction would reach an equilibrium if the reaction time is prolonged. It was evident that conversions of precursors calcined at 800 °C did not increase much after another 5-h reaction. However, precursors calcined at 600 °C still had high activity. Among all the catalysts calcined at 600 °C, S3-600 has the best performance as its conversion can reach as high as 82% and further oxidation from benzaldehyde to benzoic acid also confirmed its excellent oxidation activity. Therefore, the activity of the manganese oxides can be linked to their specific surface property. S3-600, a Mn_3_O_4_-rich material with a small amount of Mn_2_O_3_, could generate significant amount of ${\mathrm{O}}_2^{-}$ species on the surface during oxidation that would lead to the high catalytic activity in the oxidation of alcohols. S1 and S2-600 with mainly crystalline Mn_2_O_3_ could contain the dominant lattice oxygen O^2−^, surface O^−^, or β-oxygen species on the surface for the relatively lower activity in the oxidation. It was proposed that Mn_3_O_4_ with coexistence of a mix-valence state such as Mn_2_O_3_ can create defects in the materials and contribute unique and more activity in certain redox reactions [19, 34, 35]. The defects promoted the adsorption of the oxygen as indicated by O_2_-TPD, which would enhance the catalytic activity. Furthermore, the structure and crystallinity of samples obtained were similar to what Sourav and his group reported recently [6], and the manganese oxides they made still have good performance in benzyl alcohol oxidation after being recycled four times. So we believe that our catalysts could also have excellent reusability.

## Conclusions

This research aimed to tune the precursors and calcination temperature to synthesize manganese oxide nanocatalysts for the aerobic oxidation of benzyl alcohol in the liquid phase with molecular oxygen. Three groups of samples have been synthesized by Mn source with both F127 polymer (PEO_106_–PPO_70_–PEO_106_) and DA porogen (C_6_H_3_(OH)_2_–CH_2_–CH_2_–NH_2_) (S1), with only DA porogen (S2), and with only F127 polymer (S3). The crystalline MnCO_3_ has been observed for all samples before calcination. After calcination at 400 °C, the S3 transformed into an amorphous phase while S1 and S2 transformed to the body-centered cubic bixbyite Mn_2_O_3_. Upon further heating to 600 and 800 °C, S1 and S2 kept their crystalline Mn_2_O_3_ structure; S3, however, changed from the amorphous state to the tetragonal crystalline structure of hausmannite Mn_3_O_4_ with the average particle size of around 20 nm. Along with the increasing of the calcination temperature, the surface area was tapering down. Especially for samples S1 and S2, their surface areas dropped down distinctly, while for S3, the increase of the calcination temperature seemed to not have much influence on the surface area. The addition of porogen (to precursors S1 and S2) had a remarkable effect on the structure formation after calcination. TEM images (Fig. 4) show regular spherical-shaped particles of S3 compared to the oval-shaped particles of S1 and S2 after calcination at 600 °C. The temperature-programmed reduction (H_2_-TPR) indicated the existence of a small amount of Mn_2_O_3_ in the Mn_3_O_4_-dominated S3-600 sample.

The compositions for the preparation of precursors had significant influence on the structure formation of the manganese oxide nanoparticles and their surface properties. S3-600 was a Mn_3_O_4_-rich material with a small amount of Mn_2_O_3_ that could generate significant amount of ${\mathrm{O}}_2^{-}$ species on the surface after adsorption of oxygen. S1 and S2-600 with mainly crystalline Mn_2_O_3_ could contain the dominant lattice oxygen O^2−^, surface O^−^, or β-oxygen species on the surface. All the prepared samples showed target selectivity for benzyl alcohol oxidation, generating benzaldehyde and benzoic, which are essential materials in the pharmaceutical and perfumery industries. No over oxidation occurred and no CO or CO_2_ was observed during the reaction. The samples calcined at 400 °C with higher surface areas did not display much oxidation activity as the conversions were quite low. The oxidation activities of the samples were not increased proportionally with the surface area but were correlated to the crystal structure and surface sites. S3-600 with the significant amount of ${\mathrm{O}}_2^{-}$ species on the surface during oxidation exhibited the highest catalytic activity in the oxidation of alcohols. S1 and S2-600 with mainly crystalline Mn_2_O_3_ could contain the dominant lattice oxygen O^2−^, surface O^−^, or β-oxygen species on the surface that led to the relatively lower activity in the oxidation.

## Additional Files

Additional file 1: Figure S1. XRD pattern of manganese precursor S1 before and after calcination. (TIF 3067 kb)
Additional file 2: Figure S2. XRD pattern of manganese precursor S2 before and after calcination. (TIF 3066 kb)
Additional file 3: Figure S3. SEM images of manganese precursor S1 before calcination and calcined at 400, 600, and 800 °C. (TIF 397 kb)
Additional file 4: Figure S4. SEM images of manganese precursor S2 before calcination and calcined at 400, 600, and 800 °C. (TIF 452 kb)
Additional file 5: Figure S5. HR-TEM images of manganese precursor S1 calcined at 600 °C. (TIF 1762 kb)
Additional file 6: Figure S6. HR-TEM images of manganese precursor S2 calcined at 600 °C. (TIF 1633 kb)
